# *Dendrobium candidum* Wall. ex Lindl. attenuates CCl_4_-induced hepatic damage in imprinting control region mice

**DOI:** 10.3892/etm.2014.1834

**Published:** 2014-07-08

**Authors:** GUI-JIE LI, PENG SUN, QIANG WANG, YU QIAN, KAI ZHU, XIN ZHAO

**Affiliations:** Department of Biological and Chemical Engineering, Chongqing University of Education, Chongqing 400067, P.R. China

**Keywords:** *Dendrobium candidum*, hepatic damage, cytokine, gene, imprinting control region mice

## Abstract

The aim of the present study was to determine the preventive effect of the traditional Chinese medicine, *Dendrobium candidum* Wall ex Lindl. (*D. candidum*), on CCl_4_-induced hepatic damage in mice. The CCl_4_-induced hepatic damage mice were treated with *D. candidum,* and the serum levels of aspartate aminotransferase (AST), alanine aminotransferase (ALT), lactate dehydrogenase (LDH), triglyceride (TG) and total cholesterol (TC) were determined. In addition, serum cytokine levels of interleukin (IL)-6, IL-12, tumor necrosis factor (TNF)-α and interferon (IFN)-γ were analyzed with kits, while liver tissues were analyzed using hematoxylin and eosin staining and reverse transcription polymerase chain reaction (RT-PCR). Furthermore, the contents of *D. candidum* were determined by nuclear magnetic resonance (NMR). *D. candidum* was demonstrated to successfully prevent hepatic damage in mice. The serum levels of AST, ALT and LDH were significantly decreased when the mice were treated with 200 and 400 mg/kg *D. candidum,* as compared with the control mice (P<0.05). The lowest enzymatic activities were exhibited in the 400 mg/kg *D. candidum* group, which produced similar results to the positive control drug, silymarin. In addition, in the 400 mg/kg *D. candidum* group, the highest levels of TG and TC were observed among the treated groups. *D. candidum*-treated groups also demonstrated reduced levels of the serum proinflammatory cytokines, IL-6, IL-12, TNF-α and IFN-γ. The sections of liver tissue examined during histopathology in the high concentration 400 mg/kg *D. candidum* group recovered well from CCl_4_ damage; however, the sections in the 200 mg/kg *D. candidum* group revealed necrosis to a more serious degree. RT-PCR analysis was conducted on inflammation-associated genes, including nuclear factor (NF)-κB, IκB-α, inducible nitric oxide synthase (iNOS) and cyclooxygenase (COX)-2, in the livers of the mice. The 400 mg/kg *D. candidum* group demonstrated significantly decreased mRNA expression levels of NF-κB, iNOS and COX-2, but an increased expression level of IκB-α when compared with the CCl_4_-treated control group. Furthermore, using NMR, 11 compounds were identified in the *D. candidum* leaf, whose functional contents may aid the preventive effect observed in the current study. Therefore, *D. candidum* may potentially contribute to the prevention of CCl_4_-induced hepatic damage *in vivo*.

## Introduction

Liver disease, which refers to damage or disease of the liver, includes hepatitis, alcoholic liver disease, fatty liver disease, liver cirrhosis and liver cancer. The majority of these diseases are able to cause liver tissue damage (1). Numerous drugs, the majority of which are chemical, are used for the treatment of hepatic damage; however, a number of these also exhibit side effects (2). Novel biological therapies have demonstrated efficacy in treating liver damage and are also safe for human administration.

*Dendrobium* is a large genus of orchids that includes *Dendrobium candidum* Wall. ex Lindl. (*D. candidum*), which is equivalent to *Dendrobium moniliforme* (L.) Sw. (3). *D. candidum* is a traditional Chinese medicinal herb that is used raw or processed to produce health care products in China (4). The plant contains water-soluble polysaccharides, phenanthrenes and numerous amino acids. In addition, high contents of chrysotoxen and erianin may participate in the inhibitory activities in liver cancer (5).

In the present study, the preventive effect of *D. candidum* on hepatic damage was determined. The serum levels of aspartate aminotransferase (AST), alanine aminotransferase (ALT), lactate dehydrogenase (LDH), triglyceride (TG) and total cholesterol (TC), as well as levels of the inflammation-associated cytokines, interleukin (IL)-6, IL-12, tumor necrosis factor (TNF)-α and interferon (IFN)-γ, were used to determine the preventive effects of *D. candidum* on CCl_4_-induced hepatic damage in imprinting control region (ICR) mice. Liver tissue histology was also performed to determine the preventive effects of *D. candidum in vivo*. The mRNA expression levels of nuclear factor (NF)-κB, IκB-α, inducible nitric oxide synthase (iNOS) and cyclooxygenase (COX)-2 in the liver tissue were also determined to further investigate the preventive effects of *D. candidum*.

## Materials and methods

### Preparation of D. candidum

*D. candidum* was purchased from Shanghai No. 1 Pharmacy Co., Ltd. (Shanghai, China), and stored at −80°C and freeze-dried to produce a powder. A 20-fold volume of boiling water was added to the powdered sample and *D. candidum* was extracted twice by stirring overnight. The aqueous extract was evaporated and concentrated using an N-1100 rotary evaporator (Eyela; Tokyo Rikakikai Co., Ltd., Tokyo, Japan).

### Induction of hepatic damage

In total, 50 male ICR mice (age, 7 weeks) were purchased from the Experimental Animal Center of Chongqing Medical University (Chongqing, China). The mice were divided into five groups of 10 mice each. The experimental design was as follows. The normal control group were administered distilled water for 14 days and a single oral dose of vehicle [0.2 ml/kg body weight (bw) olive oil]. The CCl_4_ control group received a 14-day repeated oral administration of distilled water, followed by a single administration of CCl_4_ (0.2 ml/kg bw dissolved in olive oil, 1:1, v/v) on the final day to induce hepatic damage. The two *D. candidum* groups received 200 or 400 mg/kg bw *D. candidum* extract by gavage for 14 days, and hepatic damage was induced in the same manner as the control group. Finally, the positive control group received 100 mg/kg bw silymarin dissolved in water for 14 days, with hepatic damage induced in the same manner as for the control group mice. The mice were anesthetized 24 h following the administration of CCl_4_ and sacrificed using CO_2_ (6). Blood and liver samples were collected and preserved at −70°C until required for the biological assays. Experimental protocols were approved by the Animal Ethics Committee of Chongqing Medical University.

### Serum levels of AST, ALT and LDH

Serum levels of AST and ALT were determined using the aspartate aminotransferase, aminotransferase and lactate dehydrogenase ELISA kits (Shanghai Institute of Biological Products Co., Ltd., Shanghai, China). Serum levels of LDH were also determined using a commercially available kit (Cayman Chemical Co., Ann Arbor, MI, USA).

### Serum levels of TG and TC

Serum levels of TG and TC were determined using the triglycerides reagent and the cholesterol total ELISA kits (Shanghai Institute of Biological Products Co., Ltd.), respectively.

### Analysis of inflammation-associated cytokines in the serum by enzyme-linked immunosorbent assay (ELISA)

For the serum cytokine assay, blood from the inferior vena cava was collected in a tube and centrifuged at 1,100 × g for 10 min at 4°C. The concentrations of the proinflammatory-associated cytokines, IL-6, IL-12, TNF-α and IFN-γ, were measured using ELISA, according to the manufacturer’s instructions (BioLegend ELISA MAX™ Deluxe kit; BioLegend, San Diego, CA, USA).

### Histological examination of the liver tissue

For histological investigations, the liver tissue was fixed in 10% (v/v) buffered formalin for 24 h, dehydrated in ethanol and embedded in paraffin. Subsequently, 4-μm-thick sections were prepared and stained with hematoxylin and eosin (H&E) for observation under an Olympus BX41 microscope (Olympus, Tokyo, Japan).

### Reverse transcription polymerase chain reaction (RT-PCR) of the inflammation-associated gene expression levels in the liver tissue

Total RNA from the liver tissue samples was isolated using TRIzol reagent (Invitrogen Life Technologies, Carlsbad, CA, USA), according to the manufacturer’s instructions. RNA was digested with RNase-free DNase (Roche Diagnostics, Basel, Switzerland) for 15 min at 37°C and purified using an RNeasy kit (Qiagen, Hilden, Germany), according to the manufacturer’s instructions. The cDNA was synthesized from 2 μg total RNA by incubation at 37°C for l h with avian myeloblastosis virus reverse transcriptase (GE Healthcare, Little Chalfont, UK) with random hexanucleotides, according to the manufacturer’s instructions. The sequences of primers used to specifically amplify the genes of interest were as follows: NF-κB forward, 5′-CAC TTA TGG ACA ACT ATG AGG TCT CTG G-3′ and reverse, 5′-CTG TCT TGT GGA CAA CGC AGT GGA ATT TTA GG-3′; IκB-α forward, 5′-GCT GAA GAA GGA GCG GCT ACT-3′ and reverse, 5′-TCG TAC TCC TCG TCT TTC ATG GA-3′; iNOS forward, 5′-AGA GAG ATC GGG TTC ACA-3′ and reverse, 5′-CAC AGA ACT GAG GGT ACA-3′; and COX-2 forward, 5′-TTA AAA TGA GAT TGT CCG AA-3′ and reverse, 5′-AGA TCA CCT CTG CCT GAG TA-3′. Glyceraldehyde 3-phosphate dehydrogenase (GAPDH) was amplified as an internal control gene with the following primers: Forward, 5′-CGG AGT CAA CGG ATT TGG TC-3′ and reverse, 5′-AGC CTT CTC CAT GGT CGT GA-3′. Amplification was performed in a thermal cycler (Eppendorf, Hamburg, Germany). The PCR products were separated in 1.0% agarose gels and visualized with ethidium bromide staining (7). Expression levels were analyzed with ImageJ 1.44 software (National Institutes of Health, Bethesda, MD, USA). The computing formula was as follows: Fold ratio = gene expression/GAPDH × control numerical value (control fold ratio, 1).

### Component analysis by nuclear magnetic resonance (NMR)

Dried *D. candidum* was refluxed and extracted three times with 10 times the amount of ethyl acetate. The ethyl acetate extract was obtained after 1 h for every reflux extraction and vacuum concentration extraction. The total ethyl acetate extract was extracted by anhydrous ethanol three times. The ethanol extract was suspended in water and extracted by petroleum ether, chloroform and butanol extraction, respectively. The ethyl acetate extract was treated by gradient elution in a silica gel column with a petroleum ether-ethyl acetate system. Subsequently, the chloroform extract was treated by gradient elution in a silica gel column with a petroleum chloroform-methanol system. The butanol extract was dissolved in water that had been treated with ultrasonication, and the extraction solution was obtained following filtering. The extract was eluted using an HP2MGL macroporous resin column with water and 10, 30 and 60% ethanol. Following elution, the various solvents obtained different compounds; thus, their composition was able to be identified by NMR (Varian INOVA 400; Varian Medical Systems, Inc., Palo Alto, CA, USA). NMR was set at a ^1^H frequency of 300 MHz, a temperature of 25°C, a pulse length of 8 μsec, a spin speed of 20 Hz and scanned 64 times. The ^1^H-NMR spectra were recorded using a standard high-resolution magic angle spinning probe with magic-angle gradient.

### Statistical analysis

Data are presented as the mean ± standard deviation. Differences between the mean values for individual groups were assessed with one-way analysis of variance and Duncan’s multiple range test, where P<0.05 was considered to indicate a statistically significant difference. SAS version 9.1 (SAS Institute, Inc., Cary, NC, USA) was used to conduct the statistical analyses.

## Results

### Serum levels of AST, ALT and LDH

Serum levels of AST, ALT and LDH in the mice treated with the two concentrations of *D. candidum* were higher compared with those in the mice in the normal group (Table I). Serum levels of AST, ALT and LDH were highest in the CCl_4_-treated mice. The silymarin and *D. candidum* treatments were able to significantly reduce the serum levels of AST, ALT and LDH in mice when compared with the control treatment. Furthermore, the serum levels of AST, ALT and LDH in the mice treated with 400 mg/kg *D. candidum* were similar to those treated with silymarin. Therefore, a high concentration of 400 mg/kg *D. candidum* was able to significantly decrease the serum levels of AST, ALT and LDH in mice compared with the low concentration of 200 mg/kg *D. candidum*.

### Serum levels of TG and TC

Serum levels of TG in the mice treated with *D. candidum* were higher compared with the mice in the control group. The 400 mg/kg *D. candidum* treatment significantly increased the levels of TG in the mice and a similar level was observed to those in the normal group. Serum levels of TC in the mice in the control group were significantly lower compared with the mice treated with *D. candidum* or silymarin. The high concentration (400 mg/kg) *D. candidum*-treated group revealed the highest levels of TC among the sample groups (P<0.05; Table II).

### Proinflammatory cytokine levels in the serum

Serum levels of IL-6, IL-12, TNF-α and IFN-γ in the mice in the 200 and 400 mg/kg *D. candidum*-treated groups were significantly lower compared with the mice in the control group (Table III). However, the levels of these proinflammatory cytokines in the mice treated with 400 mg/kg *D. candidum* and silymarin were similar to those of the mice in the normal group. The levels of proinflammatory cytokines in the 400 mg/kg *D. candidum*-treated mice were lower compared with those in the 100 mg/kg silymarin-treated mice.

### Histopathological examination of the hepatic damage

**Figure 1:**
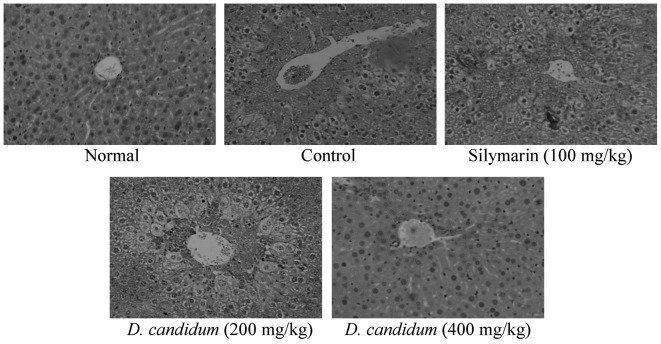
Histological images of the liver tissue of mice with CCl_4_-induced hepatic damage (magnification, ×200). *D. candidum*, *Dendrobium candidum*.

H&E staining revealed CCl_4_-induced histopathological changes in the liver tissue of the mice. Significant degeneration and necrosis of the hepatocytes were observed in the centrilobular region and perivenular inflammatory infiltrates. The histological tissue sections of the mice in the normal group demonstrated a normal morphology. However, histopathological evaluation in all the CCl_4_-treated mice revealed evidence of hepatic damage (Fig. 1). The tissue sections from the mice in the control group revealed widespread areas of congestion and hemorrhage in the centrilobular zone, as well as necrosis involving all the hepatocytes in the centrilobular zone (grade 3) (8). The 200 mg/kg *D. candidum* group demonstrated moderate congestion and hemorrhage in the area around the centrilobular vein, which extended into the midzonal cells (grade 2) (8); the majority of lobules were affected. Areas of confluent necrosis were limited to the liver cells that surrounded the centrilobular vein. The tissue sections of the mice in the 400 mg/kg *D. candidum* and silymarin groups appeared to be similar to those from the normal group (grade 1) (8). These results demonstrated that a higher concentration of *D. candidum* decreased the degree of hepatic damage in mice.

### Inflammation-associated gene expression in the liver tissues

**Figure 2:**
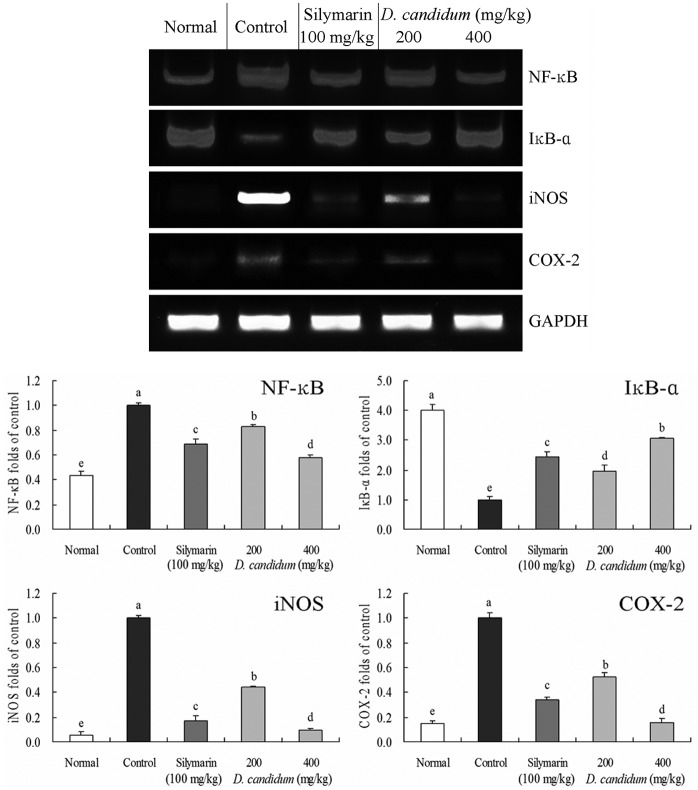
Effect of *D. candidum* on the mRNA expression levels of NF-κB, IκB-α, iNOS and COX-2 in the liver tissues of mice treated with CCl_4_. Fold ratio = gene expression/GAPDH × control numerical value (control fold ratio, 1). ^a–e^Mean values with different letters over the bars are significantly different from one another (P<0.05), according to Duncan’s multiple range test. *D. candidum, Dendrobium candidum*; NF, nuclear factor; iNOS, inducible nitric oxide synthase; COX, cyclooxygenase; GAPDH, glyceraldehyde 3-phosphate dehydrogenase.

Experiments were conducted to investigate whether the anti-inflammatory functions of *D. candidum* were associated with inhibited expression levels of the inflammation-associated genes, NF-κB, IκB-α, iNOS and COX-2. As shown in Fig. 2, the mRNA expression levels of NF-κB were reduced in the liver tissue of mice treated with *D. candidum* and silymarin when compared with the control group mice. Thus, *D. candidum* and silymarin significantly modulated the expression levels of genes associated with inflammation (P<0.05). NF-κB expression decreased, while the mRNA expression levels of IκB-α increased in the *D. candidum* and silymarin groups when compared with the control group. Furthermore, the mRNA expression levels of COX-2 and iNOS decreased in the presence of *D. candidum*, as compared with the control group, in a concentration-dependent manner. These results indicated that *D. candidum* may prevent hepatic damage by increasing anti-inflammatory activities.

### Contents of D. candidum

Following the compound assay, 11 compounds were isolated and identified in the *D. candidum* leaf. Compound 1 was obtained as a clear crystal. The ^1^H-NMR spectrum of this compound exhibited at δ 6.92 (2H, d), 6.62 (2H, d), 6.06 (2H, s), 6.03 (1H, s), 2.65 (4H, m); thus, material 1 was identified as dihydrogen resveratrol. Compound 2 was obtained as a white powder. The ^1^H-NMR spectrum of this compound exhibited at δ 6.98 (2H, d), 6.74 (2H, d), 6.62 (1H, s), 6.47 (1H, d), 4.83 (1H, d), 4.63 (1H, d), 3.1–3.8 (12H), 3.73 (3H, s), 3.69 (3H, s), 2.74 (4H, m); thus, material 2 was identified as dendromoniliside E. Compound 3 was obtained as a black-red needle. The ^1^H-NMR spectrum of this compound exhibited at δ 11.00 (1H, s), 8.15 (1H, d), 6.06 (2H, s), 8.07 (1H, d), 6.95 (1H, s), 6.83 (1H, s), 6.15 (1H, s), 3.96 (3H, s), 3.93 (3H, s); thus, material 3 was identified as denbinobin. Compound 4 was obtained as a colorless needle. The ^1^H-NMR spectrum of this compound exhibited at δ 4.72 (2H, m), 3.85 (1H, d), 6.06 (2H, s), 2.53 (1H, d), 2.49 (1H, t), 2.39 (1H, dd), 2.21 (1H, dd), 1.64 (1H, m), 1.35 (3H, s), 1.03 (3H, d), 0.95 (3H, d); thus, this material was identified as aduncin. Compound 5 was obtained as a white needle. The ^1^H-NMR spectrum of this compound exhibited at δ 8.25 (1H, s), 8.10 (1H, s), 5.90 (1H, d), 4.66 (1H, dd), 3.5–4.2 (4H, m); thus, this material was identified as adenosine. Compound 6 was obtained as a white powder. The ^1^H-NMR spectrum of this compound exhibited at δ 7.95 (1H, d), 5.85 (1H, d), 5.66 (1H, d), 3.2–4.3 (5H, m); thus, this material was identified as uridine. Compound 7 was obtained as a clear crystal. The ^1^H-NMR spectrum of this compound exhibited at δ 10.60 (1H, s), 7.92 (1H, s), 6.45 (2H, s), 5.66 (1H, d), 3.4–4.4 (5H, m); thus, the material was identified as guanosine. Compound 8 was obtained as a white powder. The ^1^H-NMR spectrum of this compound exhibited at δ 7.65 (1H, d), 7.41 (2H, d), 6.85 (2H, d), 6.33 (1H, d), 4.17 (2H, t), 1.69 (2H, m), 1.25 (54H, m), 0.85 (3H, t); thus, this material was identified as defuscin. Compound 9 was obtained as a white powder. The ^1^H-NMR spectrum of this compound exhibited at δ 7.45 (2H, d), 6.82 (2H, d), 6.81 (1H, d), 5.83 (1H, d), 4.16 (2H, t), 1.67 (2H, m), 1.23 (54H, m), 0.88 (3H, t); thus, material 9 was identified as n-triacontyl cis-p-coumarate. Compound 10 was obtained as a white powder. The ^1^H-NMR spectrum of this compound exhibited at δ 2.35 (2H, t), 1.62 (2H, m), 1.25 (24H, m), 0.88 (3H, t); thus, the material was identified as hexadecanoic acid. Compound 11 was obtained as a white powder. The ^1^H-NMR spectrum of this compound exhibited at δ 3.85 (2H, t), 1.75 (2H, m), 1.45 (2H, m), 1.22 (54H, m), 0.85 (3H, t); thus, the material was identified as hentriacontane.

## Discussion

Although *D. candidum* has traditionally been used in Chinese medicine, a limited number of studies have been published investigating its therapeutic effects. *D. candidum* has been revealed to exhibit various therapeutic effects on numerous pathological conditions, including inflammation, immunity, hyperglycemia and cancer (9).

AST and ALT are liver enzymes that are released into the general circulation following cell injury. AST is also located in numerous body tissues, including the heart, muscle, kidney, brain and lungs. ALT is located primarily in the liver, with lower quantities in the kidneys, heart and skeletal muscles (10). LDH is an enzyme located in a number of body tissues, including the liver. Increased levels of LDH have been shown to indicate liver damage. A previous study revealed that serum levels of AST and ALT in CCl_4_-treated rats were markedly increased compared with rats in the normal group, which indicated that liver damage was significantly induced by CCl_4_ (11). Low levels of TG and TC are usually observed in chronic liver diseases (12). Ghadir *et al* demonstrated a marked decrease in the plasma levels of TG and TC in patients with severe hepatitis and hepatic failure, as a result of decreased lipoprotein biosynthesis (13). From the serum level results in the present study, *D. candidum* appeared to exhibit preventive effects on hepatic damage.

Patients with inflammatory diseases exhibit elevated levels of serum cytokines, including IL-6, IL-12 and TNF-α, when compared with healthy individuals (14). Cytokine receptors and the inflammatory cytokines, IL-6, IL-12, TNF-α and IFN-γ, play pathogenic roles in gastric disease. Lower levels of these cytokines indicate an improved preventive effect on gastric ulcers (15,16). IL-6 functions as a proinflammatory and anti-inflammatory cytokine, and is encoded by the IL6 gene in humans (17). IL-6 is secreted by T cells and macrophages to stimulate an immune response, particularly during tissue damage, which leads to inflammation (18). IL-12 contributes to eradicating inflammation via the IFN-γ-dependent induction of the antiangiogenic factors, IFN-inducible protein 10 and monokine induced by IFN-γ (19). TNF-α is involved in systemic inflammation and is a member of a group of cytokines that induce the acute phase reaction (20). In a previous study (21), the colonic levels of IL-6, IL-12, TNF-α and IFN-γ in mice with reserpine-induced gastric ulcers were markedly decreased following *D. candidum* treatment. Based on the observations of this previous study, *D. candidum* was revealed to successfully prevent hepatic damage, with higher concentrations increasing the preventive effect.

Histopathology is an important clinical technique for diagnosing hepatic damage (22). Histopathological analysis of rat liver sections has been revealed as an effective method for evaluating hepatoprotective activity in a CCl_4_-induced mouse hepatic damage model (19). From the liver tissue sections examined in the current study, *D. candidum* was shown to exert a preventive effect on CCl_4_-induced hepatic damage.

NF-κB, IκB-α, COX-2 and iNOS genes may be used as biomarkers to monitor visceral damage. NF-κB is a highly ubiquitous transcription factor that regulates the expression levels of genes required for cellular proliferation, inflammatory responses and cell adhesion (23). NF-κB is present in the cytosol where it is bound to the inhibitory protein, IκB. Following induction by a number of agents, NF-κB is released from IκB and translocated to the nucleus where it binds to the κB binding sites in the promoter regions of target genes (24). Following inflammatory stimulation, COX-2 and iNOS have been observed to cause deleterious effects in the liver (6). The iNOS and COX-2 enzymes may enhance inflammatory responses in the early stages (25). Inflammatory processes are mediated by multiple molecular mechanisms, and the two most prominent are the production of iNOS and COX-2. Inflammatory stimuli elicit the synthesis of iNOS and COX-2 proteins with similar time courses, indicating that the two systems are able to interact (26). Thus, *D. candidum,* as a unique polysaccharide, may contribute to the efficacy of hepatic damage and inflammation prevention.

Resveratrol is recommended in specific cases to prevent inflammation, since the compound is known to function as an antioxidant that combats colonic inflammation (27). Aduncin, a special component that has only been identified in *Dendrobium,* may exhibit an anti-inflammatory effect (28), although its functional effects require further investigation. Adenosine has pro- and anti-inflammatory effects and targets inflammatory and resident immune cells, as well as antioxidant enzymes (29). Uridine was observed to affect the levels of TNF in rats with lung inflammation, and exhibit anti-inflammatory effects *in vivo* (30). Defuscin, n-triacontyl cis-p-coumarate, hexadecanoic acid and hentriacontane have also revealed numerous functional activities for human health treatments (31). These compounds exhibit anti-inflammatory effects, which may be the reason for the effective prevention of hepatic damage by *D. candidum*.

In conclusion, the present study demonstrated the preventive effect of *D. candidum* on hepatic damage using a variety of *in vivo* experimental methods, including serum assays of AST, ALT, LDH, TG and TC levels, serum cytokine assays of IL-6, IL-12, TNF-α and IFN-γ levels, histological examinations and a liver tissue RT-PCR assay for analyzing the levels of the inflammatory-associated genes, NF-κB, IκB-α, iNOS and COX-2. Analysis of the liver tissue revealed that *D. candidum* treatment prevented CCl_4_-induced hepatic damage in mice, indicating that *D. candidum* represents a potentially useful therapeutic strategy for the treatment or prevention of hepatic damage *in vivo*.

## Figures and Tables

**Table I:** Serum levels of AST, ALT and LDH in mice following CCl_4_-induced hepatic damage.

Treatment	AST (IU/l)	ALT (IU/l)	LDH (IU/l)
Normal (untreated)	241.3±36.1^a^	118.2±17.8^a^	1432.5±201.4^a^
Control (CCl_4_-treated)	1877.6±124.7^b^	1477.6±146.1^b^	6129.0±238.5^b^
Silymarin (100 mg/kg)	521.0±22.6^c^	522.3±29.3^c^	2782.3±175.2^c^
*D. candidum* (mg/kg)			
200	779.3±34.8^d^	924.9±44.8^d^	5217.7±241.2^d^
400	451.2±23.9^e^	427.9±23.0^e^	2126.3±146.2^e^

a P<0.05, vs. normal;

b P<0.05, vs. control;

c P<0.05, vs. 100 mg/kg *D. candidum*;

d P<0.05, vs. 200 mg/kg silymarin;

e P<0.05, vs. 400 mg/kg *D. candidum*.

AST, aminotransferase; ALT, alanine aminotransferase; LDH, lactate dehydrogenase; *D. candidum*, *Dendrobium candidum*.

**Table II:** Serum levels of TG and TC in mice following CCl_4_-induced hepatic damage.

Treatment	TG (mg/dl)	TC (mg/dl)
Normal (untreated)	83.2±4.3^a^	125.3±21.8^a^
Control (CCl_4_-treated)	59.7±5.5^b^	51.3±4.5^b^
Silymarin (100 mg/kg)	67.3±3.6^c^	96.3±7.3^c^
*D. candidum* (mg/kg)		
200	63.5±2.3^d^	72.8±6.7^d^
400	74.8±3.7^e^	108.2±6.0^e^

a P<0.05, vs. normal;

b P<0.05, vs. control;

c P<0.05, vs. 100 mg/kg *D.candidum*;

d P<0.05, vs. 200 mg/kg silymarin;

e P<0.05, vs. 400 mg/kg *D. candidum*.

TG, triglyceride; TC, total cholesterol; *D. candidum*, *Dendrobium candidum*.

**Table III:** Levels of IL-6, IL-12, TNF-α and IFN-γ in mice following CCl_4_-induced hepatic damage.

Treatment	IL-6 (pg/ml)	IL-12 (pg/ml)	TNF-α (pg/ml)	IFN-γ (pg/ml)
Normal (untreated)	42.1±2.7^a^	221.2±26.3^a^	33.6±3.2^a^	24.3±5.5^a^
Control (CCl_4_-treated)	226.2±18.9^b^	764.4±34.2^b^	86.1±7.4^b^	83.2±6.3^b^
Silymarin (100 mg/kg)	81.2±11.8^c^	452.8±31.2^c^	58.2±2.8^c^	46.2±3.5^c^
*D. candidum* (mg/kg)				
200	146.7±27.4^d^	612.8±21.7^d^	71.4±1.5^d^	66.7±3.3^d^
400	64.8±5.2^e^	367.3±19.2^e^	50.6±1.1^e^	37.2±1.4^e^

a P<0.05, vs. normal;

b P<0.05, vs. control;

c P<0.05, vs. 100 mg/kg *D. candidum*;

d P<0.05, vs. 200 mg/kg silymarin;

e P<0.05, vs. 400 mg/kg *D. candidum*.

IL, interleukin; TNF, tumor necrosis factor; IFN, interferon ; *D. candidum*, *Dendrobium candidum*.
